# The efficacy of Herceptin therapies is influenced by the expression of other erbB receptors, their ligands and the activation of downstream signalling proteins

**DOI:** 10.1038/sj.bjc.6602090

**Published:** 2004-08-10

**Authors:** B L Smith, D Chin, W Maltzman, K Crosby, G N Hortobagyi, S S Bacus

**Affiliations:** 1 Cell Signaling Technology, 166B Cummings Center, Beverly, MA 01915, USA; 2 Targeted Molecular Diagnostics, Inc., 610 Oakmont Lane, Westmont, IL 6055, USA; 3 University of Texas MD Anderson Cancer Center, 1515 Holcombe Blvd, Unit 424, Houston, TX 77030, USA

**Keywords:** biomarkers, breast cancer, Herceptin

## Abstract

ErbB2 and EGFR are attractive oncology therapeutic targets as their overexpression in tumors predicts a poorer clinical outcome in a variety of epithelial malignancies. However, clinical results with therapeutic compounds targeting these receptors have been mixed. Therefore, there is a need for improved predictive biomarkers for these targeted therapeutics. In this study we analysed tissue microarrays of patients treated with combination chemotherapy and Herceptin for expression or phosphorylation of signalling proteins associated with erbB receptors to identify protein biomarkers that are predictive of breast cancer patient response. A comparison of expression or phosphorylation of these markers with patient outcome revealed that response to Herceptin depended not only on expression levels of erbB2 but also on expression of EGFR, expression of erbB ligands, expression of other receptors and phosphorylation of downstream proteins. Elucidating the biological effects of EGFR/erbB2 targeted therapeutics will enable patient tumor profiling to identify likely responders and the determination of biologically effective doses that allows chronic administration of these agents in order to maximise efficacy.

The erbB family of receptors, namely EGFR and ErbB2, are important drug targets for cancer therapeutics and are the focus of a large number of current clinical trials. In addition, one of the first approved targeted cancer therapeutics was Herceptin, an antibody inhibitor of ErbB2. The successful clinical development of Herceptin depended upon the selection of patients based upon the overexpression of ErbB2. However, the response rate for Herceptin in breast cancer, when used in combination with chemotherapy, was approximately 50% in the pivotal clinical trial (Hortobagyi, 2001). Importantly, this response rate is observed in patients that are overexpressing ErbB2. Based upon these response rates, it is clear that better predictive markers are needed to select patients for treatment with Herceptin or other erbB inhibitors.

The response rate observed with erbB inhibitors in patients selected solely on the basis of the overexpression of a single erbB receptor is not unexpected given the complexity of erbB signalling. Most tumors of epithelial origin express multiple erbB (HER) receptors and co-express one or more EGF-related ligands, suggesting that autocrine receptor activation plays a role in tumor cellular proliferation. As these ligands activate different erbB receptors, it is possible that multiple erbB receptor combinations might be active in a tumor, a characteristic that could influence its response to an erbB-targeted therapeutic (Karunagaran *et al*, 1996). The ligands present may select the dimerization partners, and may also influence the time course of membrane translocation, activation and internalization of the receptor (Peles *et al*, 1993; Tzahar *et al*, 1994; Pinkas-Kramarski *et al*, 1996). Downstream signalling may be determined by the set of docking proteins that may bind to the activated receptors. For example, erbB3 contains six major docking sites for phosphoinositide-3-kinase (PI3K). NDF/Heregulin ligand stimulation of erbB receptors causes activation of the PI3K pathway and phosphorylation of AKT (Altiok *et al*, 1999; Liu *et al*, 1999; Xing *et al*, 2000). These observations implicate PI3K/AKT in the signalling cascade that results from erbB3 heterodimerisation with overexpressed ErbB2 in breast cancer cells. Importantly, activation of PI3K/AKT promotes cell survival and enhanced tumor aggressiveness (Bacus *et al*, 2002) and inhibition of the AKT pathway may be required for Herceptin effect (Yakes *et al*, 2002). Recent results have suggested that erbB receptors may be transactivated by other receptor classes such as G protein coupled receptors, cytokine receptors and insulin-like growth factor receptor (IGF-IR) (Gschwind *et al*, 2001).

To address the need for predictive biomarkers for Herceptin response in breast cancer patients, we analysed breast cancer tissue sections taken from patients treated with Herceptin and chemotherapy by immunohistochemistry (IHC) for expression of erbB ligands and receptors and phosphorylation of downstream signalling proteins. For this analysis we used tissue microarrays of the samples. Tissue microarrays are a well-validated method to rapidly screen multiple tissue samples under uniform staining and scoring conditions (Hoos *et al*, 2001). The results of the analysis identify a set of biomarkers that best predict patient outcome following Herceptin combination therapies. Patient probability of response ranging from 0 to 100% was observed based upon the expression or phosphorylation of a small set of ligands, receptors and signalling proteins.

## MATERIALS AND METHODS

Tissue micro-arrays of 250 metastatic breast cancer patients who received first-line chemotherapy together with Herceptin were obtained from Clinomics Biosciences (Pittsfield, MA, USA). All of the tissues were obtained under institutional IRB approval. The histology of the tumors varied with infiltrating ductal carcinoma being most common. All patients received radiotherapy postsurgery. The tissue samples in the array were taken before treatment and were all taken from the primary tumor unless otherwise noted. ErbB2 expression had been determined by the Herceptest on the original biopsies for all patients. Patient response was based upon the case histories at last follow-up as decided by the independent investigators for the clinical trials from which the samples were obtained. Patients who were free of disease at the time of examination after therapy were classified as disease free. Patients whose tumors had not progressed at the time of examination were classified as having stable disease. Patients who were disease free or had stable disease were grouped together as nonprogressors. Patients who redeveloped the disease after therapy or whose tumors progressed were classified as progressors.

EGFR and erbB2 immunostaining was performed by using the prediluted EGFR and erbB2 antibodies from Ventana Medical Instruments, Inc. (VMSI, Tucson, AZ, USA). erbB3 (1 : 10) and Heregulin (1 : 25) antibodies were obtained from NeoMarkers (Fremont, CA, USA). TGF-*α* and IGF-IR antibodies were obtained from Oncogene Sciences (San Diego, CA, USA) and NeoMarkers (Fremont, CA, USA), respectively. EGFR, ErbB2, erbB3, IGF-IR, Heregulin and TGF-*α* were immunostained using the ‘BenchMark’ (VMSI) with I-VIEW (VMSI) detection chemistry. Phospho-specific HER2 (p-HER2, Y1248), phospho-specific ERK (p-ERK), phospho-AKT (p-AKT) and phospho-S6 ribosomal protein (p-S6) antibodies were obtained from Cell Signalling Technology (Beverly, MA, USA), and immunostained using a labelled streptavidin peroxidase technique. Slides for p-S6 ribosomal protein, p-ERK and p-AKT were processed with antigen retrieval using 0.1 M citrate buffer, pH 6.0 in the ‘decloaker’ (Biocare Corp.) and the sections incubated overnight with the primary antibodies at 4°C. The next day, the slides were placed onto the Autostainer (Dako Corp.) and the ‘LSAB2 kit (Dako) was used as the detection chemistry. DAB (Dako) was used as the chromagen. Slides for p-HER2 were processed with antigen retrieval using 1 mM EDTA, pH 8.0 solution and processed manually using the Vector Elite detection system. After immunostaining, all slides were counterstained manually with 4% ethyl green (Sigma). ErbB2, EGFR, erbB3, IGF-IR, TGF-*α*, Heregulin, p-ERK, p-AKT and p-S6 ribosomal protein or phosphorylation levels were quantified using alkaline phosphatase or peroxidase techniques and microscope-based image analysis of immunohistochemical stained slides (Bacus *et al*, 1997). Quantification was by means of a CAS 200 image analyser, as previously described (Bacus and Ruby, 1993). Slides for p-HER2 were scored manually following the criteria of the Herceptest. For the purpose of the analysis, tumors were classified as negative or positive for all antibodies based upon the level of staining. Statistical analysis was performed to quantify frequencies and calculate Pearson *χ*^2^ tests of significance for interactions between variables. Comparisons were performed only on samples for which all relevant data was available.

## RESULTS

### Analysis of breast cancer tissue arrays

From the original tissue array of 250 patients, A total of 75 samples were not used in this analysis for lack of clinical data or because the sections did not contain tumor tissue. Of the remaining 175 patients, 28 samples lacked Herceptest results and were also excluded from further analysis. In total, 70 patients were removed from the analysis because of low ErbB2 expression (see below). Of the remaining 77 patients, 73 were taken from the primary tumor, three from lymph nodes and one from an adrenal metastases. The demographics of these patients are presented in Table 1

**Table 1:** Demographics of breast cancer patient samples used in final analysis of tissue microarray

	**Number of patients**
Patients included in final analysis	77
*Pathology*	
Infiltrating ductal carcinoma	65
Lobular carcinoma	3
Medullary carcinoma	1
Metastatic breast carcinoma	4
Scirrhous carcinoma	2
	
*Treatment (plus Herceptin)*	
Anthracycline plus cyclophosphamide	63
Doxorubicin	14

Analysis on tissue array samples for which clinical and Herceptest data were available and who overexpressed ErbB2.

. The great majority of patients had infiltrating ductal carcinomas and received anthracycline plus cyclophosphamide. All patients had 4 mg/kg Herceptin loading dosage and 2 mg/kg weekly maintenance dosage. Overall, 15% of the patients were disease free or had stable disease while 85% had re-occurrence or progression of disease as determined at the last examination.

ErbB2 expression levels were also analysed using the arrays (results not shown) and compared to the reported Herceptest results. Our results obtained with the arrays were very similar to the Herceptest results. Therefore, in this analysis we used the Herceptest results obtained from the original sections. In total, 70 patients had 2+ or less staining intensity whereas 77 had +3 ErbB2 staining (these patients had been given Herceptin based upon a serum assay for ErbB2 expression). As expected, we observed ErbB2 expression strongly correlated with patient response; 100% of the 0 or 1+ ErbB2 patients progressed while only 77% of the 3+ patients progressed. This response rate is lower than what has been reported previously (Baselga, 2002). Based upon these results, we concentrated our analysis of biomarkers on patients that expressed erbB2 at the highest or +3 level, as these are the patients most likely to respond to Herceptin.

**Figure 1:**
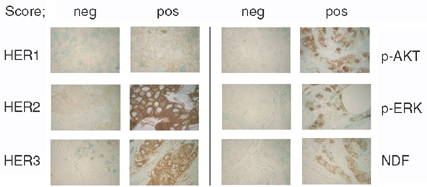
Representative images of IHC results obtained from breast cancer patient samples arrayed in a tissue microarray.

The 77 patients who overexpressed ErbB2 at the 3+ level were analysed for expression levels of p-HER2, EGFR, erbB3, IGF-IR, NDF/Heregulin, and TGF-*α* as well as activated downstream signals p-ERK and p-AKT (phosphorylated forms of ERK and AKT) and the downstream signal, p-S6 (or phosphorylated S6 ribosomal protein). Representative immunohistochemical results are presented in Figure 1

.

Similar to ErbB2, EGFR expression significantly correlated with patient outcome (Table 2

**Table 2:** Receptor tyrosine kinase expression *vs* patient outcome

**Patient group**	***n***	**% Nonprogressors**	**% Progressors**	***χ*^2^ value/*P*-value**
EGFR positive	43	30	70	3.97/0.05
EGFR negative	23	9	91	
Total	66	23	77	
				
erbB3 positive	70	29	71	0.62/NS
erbB3 negative	7	43	57	
Total	77	30	70	
				
p-erbB2 positive	17	23	77	0.42/NS
p-erbB2 negative	60	32	68	
Total	77	30	70	
				
IGF-IR positive	33	24	76	1.93/NS
IGF-IR negative	35	40	60	
Total	68	32	68	

Analysis on tissue array samples for which clinical and Herceptest data were available and who overexpressed ErbB2 at the 3+ level.

). Among these Herceptin-treated patients, the percentage of nonprogressing patients was 30% for EGFR-positive patients and 9% for EGFR-negative patients as compared to 23% for the total group of patients. erbB3 is thought to play an important role in downstream erbB signalling in that is has PI-3-Kinase docking sites and forms active heterodimers with the other erbB receptors. Among the 77 patients, 70 of them expressed erbB3. ErbB3 expression did not significantly correlate with patient outcome, p-AKT level or NDF expression although the low number of erbB3 negative patients limits these comparisons in this data set. Interestingly, p-HER2 was only observed in 22% of the patients. Of these, only 23% occurred in patients that were nonprogressors. The expression of other growth factor receptors may mediate patient response as well, either through direct stimulation of downstream pathways or through transactivation of the erbB receptors. We observed high IGF-IR expression in approximately half of the patients. IGF-IR expression alone did not correlate with patient outcome.

We found expression of erbB ligands, including NDF and TGF-*α* also varied among patients (Table 3

**Table 3:** Receptor tyrosine kinase ligand expression *vs* patient outcome following therapy

**Patient group**	***n***	**% Nonprogressors**	**% Progressors**	***χ*^2^ value/*P*-value**
NDF positive	55	39	62	6.35/0.02
NDF negative	22	9	91	
Total	77	30	70	
				
TGF-*α* positive	38	34	66	0.34/NS
TGF-*α* negative	29	28	72	
Total	67	31	69	

Analysis on tissue array samples for which clinical and Herceptest data were available and who overexpressed ErbB2 at the 3+ level.

). Approximately 70% of the patients expressed high levels of NDF while approximately 57% expressed high levels of TGF-*α*. A significant correlation was observed between NDF levels and patient outcome. A very high proportion of NDF-negative patients progressed (91%) whereas 62% of NDF-positive patients progressed compared to a 70% progression rate in the total patient group. No predictive relationship was observed between TGF-*α* levels and patient outcome (Table 3). However, the combination of TGF-*α* or NDF expression and EGFR overexpression did positively correlate with patient outcome (data not shown; *P*=0.02 and 0.03, respectively).

The activation of heterodimers of erbB2 with erbB3 and EGFR results in activation of the ERK and PI3K/AKT pathways. Comparison of the levels of activated or phosphorylated ERK alone failed to demonstrate any significant effect of elevated p-ERK levels as a factor for patient outcome. Similarly, AKT activation (p-AKT) or phosphorylation of S6 ribosomal protein alone, which integrates multiple signals through mTOR and p70 S6 kinase, did not significantly correlate with patient outcome.

To increase the predictive power of our analysis, we next considered an analysis in which two or more of these biomarkers were combined to characterise the tumor. In this analysis we found that the combination of low EGFR expression and high ERK activation significantly predicted a poor outcome (Table 4

**Table 4:** Analysis of receptor and downstream protein activation or ligand expression *vs* outcome in patients following therapy

**Patient group**	***n***	**% Nonprogressors**	**% Progressors**	***χ*^2^ value/*P*-value**
EGFR pos/p-ERK pos	21	14	86	8.55/0.05
EGFR pos/p-ERK neg	19	42	58	
EGFR neg/p-ERK pos	9	0	100	
EGFR neg/p-ERK neg	14	14	86	
Total	63	21	79	
				
EGFR pos/p-AKT pos	17	18	82	6.96/NS
EGFR pos/p-AKT neg	26	38	62	
EGFR neg/p-AKT pos	5	20	80	
EGFR neg/p-AKT neg	18	6	94	
Total	66	23	77	
				
IGF-IR pos/p-S6 pos	13	8	92	10.54/0.02
IGF-IR pos/p-S6 neg	20	35	65	
IGF-IR neg/p-S6 pos	12	67	33	
IGF-IR neg/p-S6 neg	23	26	74	
Total	68	32	68	
				
EGFR pos/NDF pos	31	39	61	8.94/0.05
EGFR pos/NDF neg	12	8	92	
EGFR neg/NDF pos	16	12	88	
EGFR neg/NDF neg	7	0	100	
Total	66	23	77	

Analysis on tissue array samples for which clinical and Herceptest data were available and who overexpressed ErbB2 at the 3+ level.

). A comparison combining high EGFR and high p-AKT predicted a poor patient outcome as well (18 *vs* 38% for patients with low p-AKT levels). The combination of high EGFR and high NDF or TGF-*α* expression predicted a better outcome compared to patients that had low expression of EGFR and the ligand. For example, while 39% of the patients with high EGFR and NDF expression did not progress, all of the patients with low EGFR and NDF expression progressed (Table 4) compared to 23% nonprogressors in the total patient group.

The combination of low IGF-IR expression and high S6 ribosomal protein phosphorylation gave a favorable patient response outcome (67% nonprogressors, Table 4). This compares to patients with high IGF-IR expression and high S6 ribosomal protein phosphorylation, 8% of who were nonprogressors. The best combination of markers for the prediction of patient response was NDF, IGF-IR and p-S6 (Table 5

**Table 5:** Analysis of ligand and receptor expression and downstream protein activation *vs* patient outcome in patients following therapy

**Patient group**	***n***	**% Nonprogressors**	**% Progressors**	***χ*^2^ value/*P*-value**
NDF neg/p-S6 pos/IGF-IR neg	2	50	50	19.41/0.01
NDF neg/p-S6 neg/IGF-IR neg	9	11	89	
NDF neg/p-S6 neg/IGF-IR pos	4	0	100	
NDF neg/p-S6 pos/IGF-IR pos	4	0	100	
NDF pos/p-S6 pos/IGF-IR neg	7	100	0	
NDF pos/p-S6 neg/IGF-IR pos	16	44	56	
NDF pos/p-S6 neg/IGF-IR neg	14	36	64	
Total	56	37	63	
				
NDF neg/p-ERK pos/EGFR neg	3	0	100	12.75/NS
NDF neg/p-ERK neg/EGFR neg	4	0	100	
NDF neg/p-ERK neg/EGFR pos	10	20	80	
NDF neg/p-ERK pos/EGFR pos	6	0	100	
NDF pos/p-ERK pos/EGFR neg	5	0	100	
NDF pos/p-ERK neg/EGFR pos	13	54	46	
NDF pos/p-ERK neg/EGFR neg	6	17	83	
NDF pos/p-ERK pos/EGFR pos	18	28	72	
Total	65	23	77	

Analysis on tissue array samples for which clinical and Herceptest data were available and who overexpressed ErbB2 at the 3+ level.

). All of the patients who had high NDF expression, low IGF-IR expression and high S6 phosphorylation had stable disease or were disease free (however, the number of patients in this category was only seven). In comparison, all of the patients who had low NDF expression and high IGF-IR expression progressed, regardless of S6 status (Table 5). In patients with high NDF and EGFR expression levels, phosphorylation of ERK correlated with a difference in progression from 28% (high p-ERK) to 54% (low p-ERK; Table 5). Similarly, those patients with low levels of p-AKT with any other combination of biomarkers that includes the expression of NDF, did better than those that overexpress this protein (results not shown). Taken together, our data show that ErbB2 together with its ligand and other erbB receptors and ligands as well as other growth factor receptors play a role in Herceptin response. Importantly, analysis of a select combination of these proteins correlated with progression rates that varied from 0 to 100%. Therefore, these data suggest that the use of a defined set of markers may accurately predict progression. For comparison, the ‘*a priori*’ level of prediction of progression, without the use of these biomarkers, ranged from 63 to 79%

## DISCUSSION

**Figure 2:**
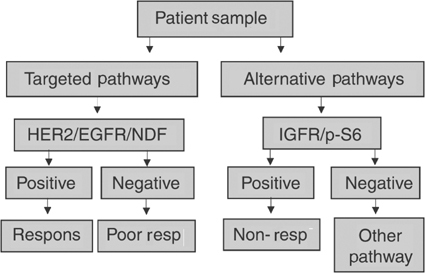
Diagnostic protocol for predicting patient response to combination Herceptin and chemotherapy.

The success of Herceptin therapies in the treatment of breast cancer patients has been limited although those patients treated overexpress the ErbB2 protein. Our results demonstrate that the status of EGFR and the erbB ligands, NDF and TGF-*α* affect Herceptin therapy response in breast cancer patients. Patients whose tumors express high levels of EGFR, ErbB2 and NDF or TGF-*α* are most likely to respond. Other studies that considered cell lines in culture have shown that indeed not all tumor cells respond to inhibition of ErbB receptors, despite exhibiting aberrant EGFR and/or ErbB2 expression (Motoyama *et al*, 2002). In this respect, it has been reported that a combination of the EGFR-directed mAb C225 and the erbB2 directed mAb 4D5 or using dual EGFR/HER2 inhibitors inhibited proliferation of tumor cell lines more strongly than either mAb alone (Ye *et al*, 1999; Motoyama *et al*, 2002; Xia *et al*, 2002). A diagnostic protocol for predicting patient response to Herceptin and chemotherapy is presented in Figure 2

. The left arm of the protocol details the analysis of the targeted pathways, namely the erbB receptors and their ligands. The right arm of the protocol details the analysis of alternative pathways, namely the IGF-IR pathway and downstream signalling.

Our results suggest that the IGF-IR receptor may mediate patient response to breast cancer therapies targeting ErbB2. High IGF-IR expression combined with high S6 ribosomal protein phosphorylation correlated with poor patient response regardless of erbB expression indicating that IGF-IR was acting directly to activate downstream signalling rather than through transactivation of erbB receptors. IGF signalling in breast cancer has been shown to occur through AKT activation (Dufourny *et al*, 1997; Oh *et al*, 2002), which would lead to S6 ribosomal protein phosphorylation. Hence, S6 phosphorylation may indicate active IGF signalling in those tumors overexpressing IGF-IR. A role for IGF-IR in patient response has been suggested by cell line studies. Lu *et al*. (2001) reported that Herceptin resistance could occur through activation of IGF-IR. Other studies have indicated that co-targeting IGF-IR as well as ErbB2 would produce synergistic inhibition of growth in breast cancer cells (Camirand *et al*, 2002). Based upon our results as well as the results of these published studies, analysis of IGF-IR expression and downstream signalling may be critical for an accurate assessment of potential Herceptin response in breast cancer patients (right arm of Figure 2).

The percentage of patients that overexpress HER2 and also are positive for p-HER2 in this study is similar to the percentage observed by Thor *et al*, (2000). In addition, more of these patients progressed than had stable disease or were disease free following treatment. These observations clearly raise questions as to the phosphorylation status of HER2 in tumor samples. The failure to see more p-HER2 positive tumors and to see a correlation with drug response may be due to rapid dephosphorylation of the receptor *in vivo* or during tissue handling or inadequate sensitivity of the antibody. However, cell line studies indicate that the p-HER2 antibody is capable of detecting HER2 phosphorylation following ligand exposure (results not shown).

Our analysis of downstream signalling and patient response is complicated by the inclusion of chemo- and radiotherapy in addition to Herceptin for the patients analysed. AKT or MAP kinase pathway activation, for example, is known to play a role in response to DNA-damaging agents (Bacus *et al*, 2001; Clark *et al*, 2002). Therefore, consideration of downstream signalling in patients undergoing a combination of therapies may provide additional predictive information not available at the level of the receptor or ligand. Analysis of patients treated with Herceptin as a single agent therapy would be needed to determine which of the biomarkers we identified are mediating response to Herceptin itself *vs* the other therapies. However, our results are useful in the design of diagnostic tests for breast cancer patients undergoing the common Herceptin combination therapies. The protocol outlined in Figure 2 represents a first attempt at such a diagnostic test.
